# De Graffe and Cochrane

**Published:** 1875-05

**Authors:** 


### DE GRAFFE & COCHRANE.

A good subscriber and friend asks us why we say pleasant words so frequently for the great Furniture House at No. 152 West Twenty-third street, near Sixth avenue, the name of which appears at the head of this article, and asks, "Are theie not other furniture establishments in this great city, as good as the one which you so persistently recommend, and if so is it right that you should single out one concern and make it famous, to the disparagement of all the rest ?"

To this we have to reply, that there are, no doubt, other furniture stores as large and as well conducted as the one of which we speak. But we know little about them, while we have considerable knowledge of the ways of the house alluded to, and are quite willing to speak what we do know and testify what we have seen. We have passed through the building and have examined beautiful articles of household adornment, and have felt that if we had a thousand fine houses to furnish, we could hardly do better than to secure their outfit here. We have closely inspected numerous chairs, and sofas, and book-cases, and sideboards, and desks which were bought at this establishment last fall, and which, after having been exposed to furnace heat in a close room all winter, are as free from warp, or check, or shrinkage now as when they were taken from the factory. This is a good deal to say in these days when unseasoned wood and careless work appear to be quite common. We have seen the time when it would have been a great comfort to us if we could have been directed to such an establishment, and we doubt not that many of our readers, who have acted upon our suggestions in reference to this, have thanked us in their hearts for our advice.

Our friend errs in supposing that we recommend De Graffe & Cochrane to the disparagement of other dealers. This is very far from our thoughts or intentions. We speak of the house alluded to as we and our friends have found it, and do not, in so doing, detract from the merits of any other concern. We here repeat what we have said before, that we believe our friend will find excellent material, artistic workmanship and fair prices at the establishment alluded to.

